# Rapid Progressive Liver Atrophy During Immune Checkpoint Inhibitor Hepatitis: Imaging Perspectives Beyond Aminotransferase Elevation

**DOI:** 10.1002/ccr3.73045

**Published:** 2026-06-25

**Authors:** Chukwuka Elendu, Adetokunbo A. Adesina, Olisa S. Okabekwa, Ebube C. Mbah, Favour K. Mbakwe

**Affiliations:** ^1^ Federal University Teaching Hospital Owerri Nigeria; ^2^ Buckinghamshire NHS Trust Aylesbury UK; ^3^ University of Nigeria Teaching Hospital Enugu Nigeria; ^4^ Ahmadu Bello University Zaria Nigeria; ^5^ Obafemi Awolowo University Teaching Hospitals Complex Ile‐Ife Nigeria

**Keywords:** cirrhosis, computed tomography volumetry, immune checkpoint inhibitors, immune‐mediated hepatitis, liver atrophy

## Abstract

Severe immune checkpoint inhibitor–associated hepatitis may progress despite only modest aminotransferase elevations, particularly in patients with cirrhosis. Recognition of atypical presentations and integration of clinical, radiologic, and multidisciplinary assessment may improve risk stratification and guide timely management of clinically significant hepatic injury.

Dear Editor,

We read with great interest the recently published case report titled “Autopsy‐proven immune‐mediated hepatitis with rapid liver atrophy despite minimal aminotransferase elevation after durvalumab plus tremelimumab therapy for hepatocellular carcinoma: a case report” [1], which describes fatal immune‐mediated hepatitis characterized by progressive liver atrophy and liver failure despite only modest aminotransferase elevation in a patient with cirrhosis receiving immune checkpoint inhibitor therapy.

Immune checkpoint inhibitors, such as durvalumab plus tremelimumab, have improved outcomes in hepatocellular carcinoma (HCC) but are associated with potentially severe immune‐related adverse events, including immune‐mediated hepatitis [2, 3, 4]. Current monitoring and grading frameworks rely largely on aminotransferase elevations, particularly alanine aminotransferase (ALT) and aspartate aminotransferase (AST), alongside bilirubin and coagulation parameters [5].

The observed discordance between aminotransferase levels and clinical deterioration highlights that severe immune‐mediated hepatic injury may progress despite only modest aminotransferase elevations [1]. In cirrhosis, reduced hepatocellular reserve may limit enzyme release, causing biochemical markers to underestimate the extent of hepatocyte loss. Consequently, reliance on AST and ALT alone may fail to reflect disease severity and provide false reassurance during the progression of clinically significant liver injury [1, 6].

Serial computed tomography (CT) volumetry demonstrated a 56% reduction in liver volume that paralleled worsening jaundice, coagulopathy, ascites, and eventual liver failure despite the absence of marked transaminase elevation (Figure 1) [1]. These findings suggest that quantitative imaging may complement conventional biochemical assessment in detecting clinically significant hepatic injury. Although CT volumetry is routinely used in hepatobiliary surgery and transplantation to assess liver volume and regenerative capacity, its role in monitoring immune‐mediated liver injury remains largely unexplored [7].

**FIGURE 1:**
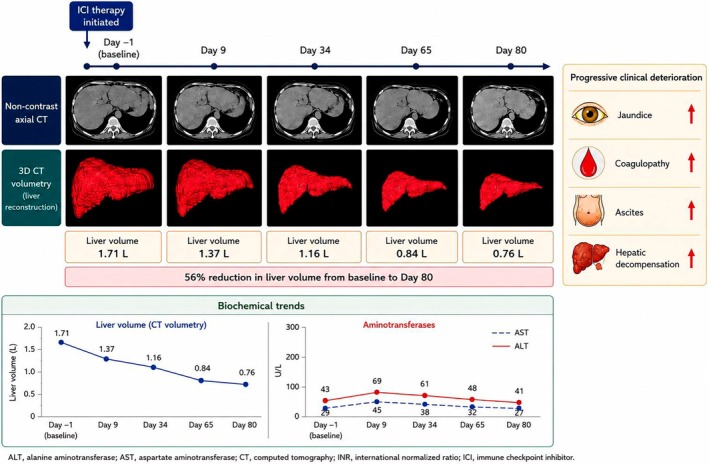
Serial CT imaging and volumetric assessment demonstrating progressive liver atrophy despite modest aminotransferase elevations. CT volumetry showed a 56% reduction in liver volume, accompanied by worsening jaundice, coagulopathy, ascites, and hepatic decompensation. Adapted from data and imaging findings reported in [1].

Liver atrophy has been associated with chronic inflammatory injury, severe drug‐induced liver injury, and acute‐on‐chronic liver failure [8]. In the setting of immune checkpoint inhibitor therapy, progressive volume loss may reflect ongoing immune‐mediated hepatic injury, suggesting that serial quantitative imaging could complement conventional biochemical monitoring.

Another important observation is the coexistence of colitis, hepatitis, and myocarditis, reflecting the multisystem nature of immune‐related adverse events [9]. Progressive liver failure despite corticosteroid treatment for colitis suggests that organ‐specific toxicities may respond differently to therapy, warranting continued surveillance for concurrent immune‐mediated injury.

The autopsy findings are particularly informative because pathological confirmation of fatal immune checkpoint inhibitor–associated hepatitis remains uncommon. Lobular hepatitis with patchy necrosis and CD8‐predominant lymphocytic infiltration supports a cytotoxic T‐cell–mediated mechanism of hepatic injury distinct from classical autoimmune hepatitis [1, 10].

These findings also highlight limitations of current laboratory‐based grading systems for immune‐mediated hepatitis in patients with cirrhosis [5]. Clinically significant deterioration, including worsening synthetic dysfunction, hepatic decompensation, and progressive liver atrophy, may occur despite relatively low toxicity grades based on aminotransferase thresholds. Incorporating bilirubin, coagulation parameters, clinical decompensation, and imaging biomarkers into assessment frameworks may improve risk stratification in this population (Figure 2) [1].

**FIGURE 2:**
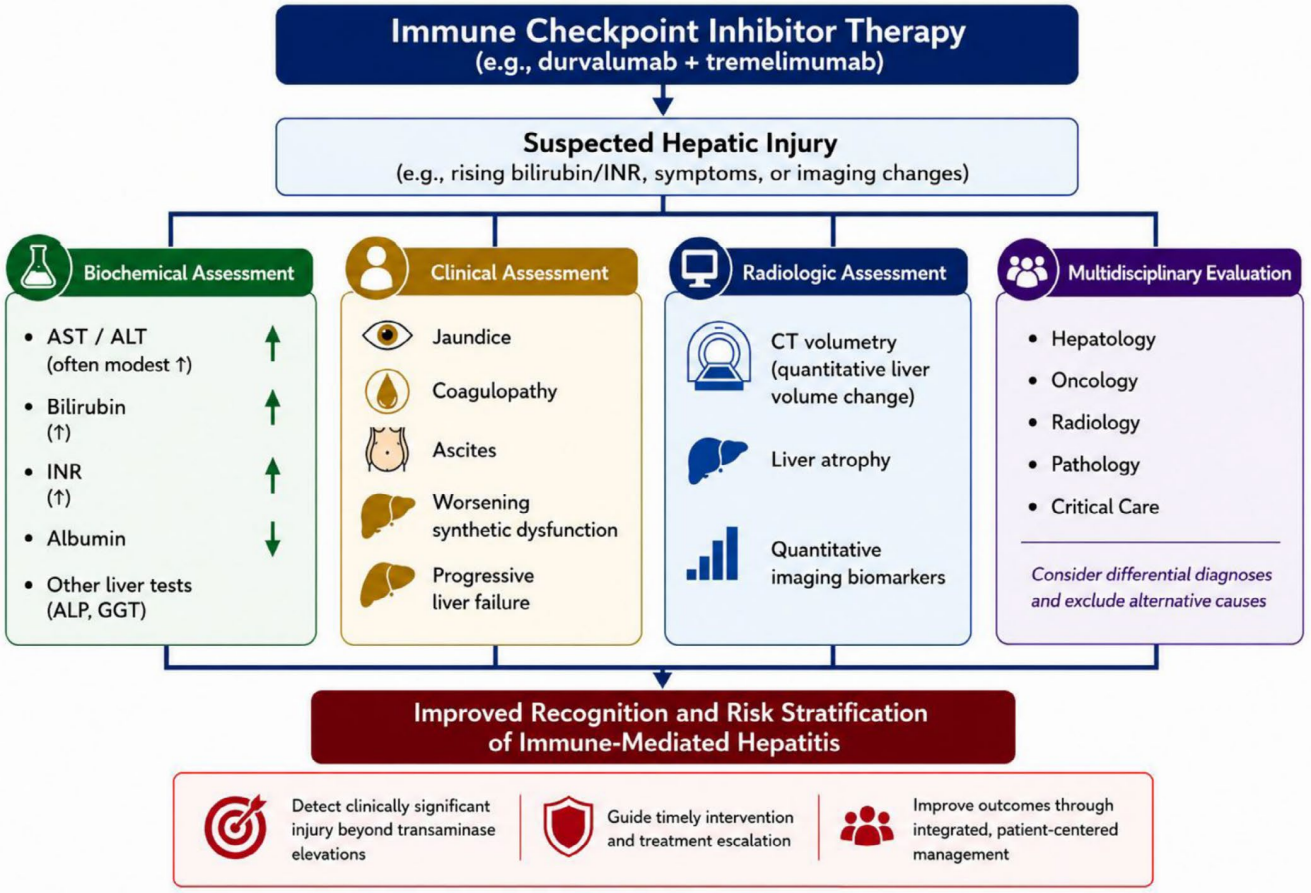
Proposed multimodal assessment framework for immune checkpoint inhibitor–associated hepatitis integrating biochemical, clinical, radiologic, and multidisciplinary evaluation to improve recognition and risk stratification of severe hepatic injury.

The implications extend beyond hepatocellular carcinoma, as immune checkpoint inhibitors are increasingly used in patients with underlying chronic liver disease, including metabolic dysfunction‐associated steatotic liver disease, chronic viral hepatitis, alcohol‐associated liver disease, and compensated cirrhosis [11]. Because these conditions may alter the biochemical expression of hepatic injury, clinicians should maintain a high index of suspicion when liver function deteriorates, even in the absence of marked aminotransferase elevations.

The importance of multidisciplinary evaluation is also evident [1], as expertise from hepatology, oncology, radiology, pathology, and critical care may facilitate recognition of atypical presentations and exclusion of alternative causes of liver dysfunction. Such collaboration can support timely diagnostic and therapeutic decisions in the management of complex immune‐related toxicities.

The progression of liver failure despite corticosteroid therapy highlights the challenges of managing immune‐mediated hepatitis. Although corticosteroids remain the mainstay of treatment, resistance is increasingly recognized and may contribute to poor outcomes, particularly when diagnosis is delayed or substantial hepatocyte loss has already occurred [12]. Further research is needed to define the role of imaging biomarkers in immune‐mediated hepatitis. Prospective studies should evaluate whether changes in liver volume and other quantitative imaging parameters correlate with disease severity, treatment response, and clinical outcomes. Given the rarity of autopsy‐confirmed cases, multicenter registries may be required to validate imaging‐based monitoring strategies.

In conclusion, aminotransferase‐based assessment may underestimate the severity of immune‐mediated hepatitis in patients with cirrhosis. Progressive liver atrophy and worsening synthetic dysfunction despite modest enzyme elevations underscore this limitation. Incorporating biochemical, clinical, and radiologic parameters into routine assessment may improve recognition of liver toxicity during immune checkpoint inhibitor therapy.

## Author Contributions

**Chukwuka Elendu:** conceptualization, data curation, investigation, validation, formal analysis, writing – original draft, writing – review and editing, visualization, supervision, project administration, methodology. **Adetokunbo A. Adesina:** investigation, validation, writing – original draft. **Olisa S. Okabekwa:** formal analysis, validation, writing – review and editing. **Ebube C. Mbah:** investigation, validation, writing – review and editing. **Favour K. Mbakwe:** supervision, writing – review and editing, validation.

## Funding

The authors have nothing to report.

## Disclosure

The views expressed in this paper are solely those of the authors and do not represent the official positions of any affiliated institutions.

## Ethics Statement

Ethical approval was not required, as this letter does not involve new patient data, human subject research, or original clinical investigation.

## Consent

Written informed consent for publication was obtained where applicable. This letter does not include new patient data.

## Conflicts of Interest

The authors declare no conflicts of interest.

## Data Availability

Data sharing not applicable to this article as no datasets were generated or analysed during the current study.
